# 53BP1: guarding the genome with a novel liquid weapon

**DOI:** 10.1038/s42003-022-03401-0

**Published:** 2022-05-10

**Authors:** Naveen Kumar Tangudu, Katherine M. Aird

**Affiliations:** Department of Pharmacology & Chemical Biology and UPMC Hillman Cancer Center, University of Pittsburgh School of Medicine, Pittsburgh, PA 15213 USA

**Keywords:** Cell biology, Biochemistry

## Abstract

In this Comment, Naveen Tangudu and Katherine Aird discuss recent findings showing that 53BP1 regulates heterochromatin through liquid-liquid phase separation.

A recent article in Nature Communications by Zhang et al. report that 53BP1 regulates of heterochromatin via liquid-liquid phase separation^1^. Here we will highlight their important observations and implications of the non-canonical function of 53BP1 in genome stability that is uncoupled from its role in DNA repair.

Heterochromatin, non-active or non-transcribed condensed DNA that is characterized by repressive epigenetic histone marks such as H3K9me2/3, is a highly dynamic chromatin domain with a critical function in numerous biological processes including DNA repair and genome stability^2^. Processes that perturb heterochromatin integrity may lead to DNA damage and genome instability, ultimately affecting cell fate^2^. The utmost responsibility of DNA damage response (DDR) signaling is to protect the genome from disturbances that arise upon dysfunctional heterochromatin, which may lead to several pathologies including cancer and aging^3^. In response to DNA damage, a signaling cascade coordinated by several proteins recognizes DNA lesions and prompts cell cycle arrest and DNA repair^4^. At sites of DNA damage, the DDR is initiated by the kinase Ataxia-Telangiectasia Mutated (ATM), phosphorylation of the histone variant H2AX (γH2AX), and recruitment of the DNA damage checkpoint protein 1 (MDC1) adaptor protein, which is followed by accumulation of the scaffold protein tumor suppressor p53-binding protein 1 (53BP1)^4^. 53BP1 is a member of the tudor-containing proteins that reads unique methylation events on histones to facilitate DNA damage repair or regulate transcription. Canonically, 53BP1 plays a crucial role in orchestrating the switch between double strand break (DSB) repair pathways by promoting non-homologous end joining (NHEJ)-mediated DSB repair while inhibiting homologous recombination (HR)^5^. Indeed, previous reports have demonstrated that 53BP1 is important for genome integrity through its DNA DSB repair function and cell cycle checkpoint signaling^5,6^. In recent years, multiple other functions of 53BP1 have been reported^7–9^; however, the mechanisms underlying the distinction between DNA DSB repair and other functions related to genome stability in unperturbed cells remained unclear. Zhang et al. report that 53BP1 regulates heterochromatin through liquid-liquid phase separation (LLPS) that is uncoupled from its role in DNA repair. Importantly, this newly defined phase separated function of 53BP1 protects cells from stress-induced DNA damage and cellular senescence, highlighting its importance in genome stability and integrity.

As a DNA repair protein, 53BP1 dimerization has been shown to promote 53BP1 recruitment at DSBs and self-association into liquid condensates^10^. In this study, the authors observed distinct nuclear 53BP1 puncta under normal growth conditions in the absence of DNA damage, uncoupling its role in DNA repair^1^. Interestingly, these distinct 53BP1 nuclear puncta co-localize with the core heterochromatin protein HP1α and the repressive epigenetic histone mark H3K9me3, a marker of constitutive heterochromatin. Knockout of 53BP1 decreased H3K9me3 puncta and the size of heterochromatin centers, indicating that 53BP1 is a critical component of heterochromatin organization and maintenance. Further analysis of the 53BP1 protein identified a core component containing the oligomerization domain (OD) that is critical for puncta formation (Fig. 1). Additionally, using genome wide ChIP-Seq, the authors correlated enrichment of 53BP1 and H3K9me3 at multiple loci, strengthening the observation that 53BP1 localizes to heterochromatin. While 53BP1 is known to be recruited to DSBs in heterochromatin^11^, this is the first report demonstrating recruitment of 53BP1 to heterochromatin under unperturbed conditions. One key function for heterochromatin in maintaining genome stability is to repress tandem repetitive DNAs or repetitive elements^12^. Upon loss of heterochromatin, these elements can cause genomic instability by affecting chromosome segregation, inducing replication stress, increasing transposon hopping, or impairing correct DSB repair^2^. Indeed, the authors found that binding of 53BP1 at heterochromatin marked by H3K9me2/3 is required to repress the expression of repetitive elements. Therefore, it is interesting to speculate that 53BP1 may function in tandem with other histone modifications to epigenetically regulate multiple other biological processes outside of its role in DNA repair.

**Fig. 1:**
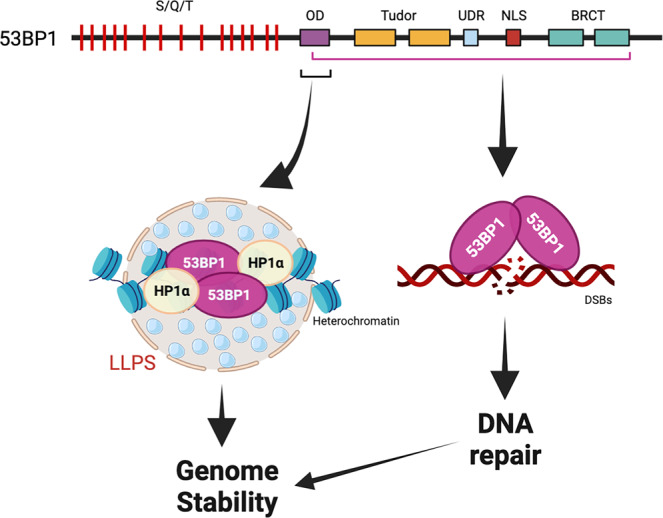
53BP1 domains that are required for genome stability through heterochromatin maintenance and double strand break repair. Schematic diagram of 53BP1 protein domains that are important for DNA repair and heterochromatin maintenance functions of 53BP1. The oligomerization domain (OD) is required to bind to HP1α and for liquid-liquid phase separation (LLPS) at heterochromatin, which promotes genome stability. Many domains of 53BP1 are necessary for recruitment of 53BP1 to DNA double strand breaks (DSBs). LLPS liquid-liquid phase separation, HP1α heterochromatin protein 1 alpha, OD oligomerization domain, Tudor tandem tudor domain, UDR ubiquitin-dependent recruitment motif, NLS nuclear localization sequence, BRCT BRCA1 carboxy terminal domain, DSBs, DNA double strand breaks. Created with BioRender.com.

The most striking finding of this study is that 53BP1 forms nuclear puncta along with HP1α to regulate heterochromatin via liquid-liquid phase separation (LLPS)^1^. Liquid-liquid phase separation has emerged as a crucial mechanism to facilitate multiple biological processes including chromatin organization^13^, DNA repair^14^, and gene transcription^15^. LLPS has been used to explain the formation of known nuclear organelles such as nucleoli and promyelocytic leukemia nuclear bodies (PML NBs) as well as other membraneless condensates (e.g., nucleosome arrays, DNA damage foci, stress granules, proteasomes, autophagosomes, etc.)^16^. Recent studies in multiple organisms, including mammalian cells, found that core heterochromatin proteins including HP1α, SUV39H1, and TRIM28 can undergo LLPS when certain conditions are met, including specific protein–protein interactions and/or post-translational modifications^17^. While 53BP1 has been previously shown to undergo phase separation^6^, prior to this study, it was unknown what factors or proteins contribute to the regulation of heterochromatin by 53BP1 through LLPS. The authors found that HP1α is required for 53BP1 puncta formation and 53BP1’s LLPS^1^. By re-expressing different truncated and mutated 53BP1 constructs, the authors further identified domains required for LLPS and puncta formation that are dispensable for 53BP1 foci formation at DNA DSBs (Fig. 1). Together, these data provide evidence that LLPS of 53BP1 and puncta formation at heterochromatin are distinct from the role of 53BP1 in DNA repair.

Finally, the authors set out to determine the contribution of 53BP1 puncta on functional readouts of genome stability. After treating cells with the DNA damage agent bleomycin, the truncated mutant 53BP1 that is still capable of puncta formation but incapable of forming foci at DSBs rescued both total DNA damage and survivial^1^. These data suggest that the LLPS-mediated 53BP1 puncta formation at heterochromatin is indeed critical for some aspects of genome stability. Genome instability due to loss of heterochromatin can also lead to cellular senescence, a state of stable cell cycle arrest with characterized hallmarks of increased DNA damage response, senescence associated heterochromatic foci (SAHF), and the senescence associated secretory phenotype (SASP)^18^. The SASP is characterized by secretion of a wide range of cytokines, chemokines, matrix metalloproteinases, and growth factors^18^. Many labs, including ours, have shown that SASP components *IL6* and *CXCL8* (IL8) are highly transcriptionally upregulated in senescent cells^19–21^. Here, the authors found that induction of *IL6* and *CXCL8* was rescued to some extent by 53BP1 constructs that are proficient in puncta formation. Interestingly, our previous work has shown that high-mobility group box 2 (HMGB2) protects SASP genes from heterochromatin to allow for their transcriptional upregulation^21^. Further, we recently found that the histone methyltransferase disruptor of telomeric silencing 1-like (DOT1L), which promotes the active histone marks H3K79me2/3, helps to promote SASP gene expression^19^. It would be interesting to determine whether 53BP1 antagonizes HMGB2 and/or DOT1L to regulate SASP genes. In addition to this, future studies to characterize other senescent markers besides the SASP, such as senescence-associated beta-galactosidase (SA-β-gal) activity, cell proliferation, cell size, and SAHF, would strengthen the notion of 53BP1’s involvement in cellular senescence through this novel mechanism.

Understanding mechanisms related to heterochromatin formation and maintenance is critically important since its dysregulation can lead to genome instability, thereby promoting pathological consequences such as cancer or aging. The study from Zhang et al. advances our knowledge of how heterochromatin is maintained to promote genome stability through a novel LLPS-mediated function of 53BP1, although additional studies to further mechanistically delineate how 53BP1 promotes heterochromatin maintenance and what effects this has on cell fate are needed. Due to its known role in promoting HR-mediated DNA repair, there has been some interest in identifying 53BP1 inhibitors to help with CRISPR/Cas9-mediated genome editing^22^. The data presented by Zhang et al. would suggest that caution is needed when designing these inhibitors. As they identified specific amino acids in the OD domain that are required for heterochromatin maintenance yet dispensable for binding of 53BP1 at DSBs, the observations presented in this study not only provide new insight into functions for 53BP1, but also provide the opportunity to target specific domains related only to DNA repair and NHEJ to inhibit this pathway while maintaining heterochromatin.
